# Frontal Cortex Entropy Derived From Resting‐State fNIRS for Brain Age Prediction in Major Depressive Disorder

**DOI:** 10.1155/da/4616978

**Published:** 2026-04-30

**Authors:** Shanling Ji, Yang Tian, Xinyu Lin, Xia Liu, Hao Yu

**Affiliations:** ^1^ School of Mental Health, Jining Medical University, Jining, Shandong Province, China, jnmc.edu.cn; ^2^ Department of Sleep Medicine, Shandong Province Daizhuang Hospital, Jining, Shandong Province, China

**Keywords:** brain age, brain entropy, fNIRS, major depressive disorder

## Abstract

**Objective:**

This study investigated the potential of frontal brain entropy (BEN) metrics derived from resting‐state functional near‐infrared spectroscopy (rs‐fNIRS) as neurophysiological biomarkers for predicting brain age in patients with major depressive disorder (MDD).

**Methods:**

Rs‐fNIRS data (26 channels) were acquired from the frontal cortex of 49 healthy controls (HCs) and 35 MDD patients. Time‐series signals for oxyhemoglobin (HbO), deoxyhemoglobin (HbR), and total hemoglobin (HbT) were extracted. Static BEN was computed as permutation entropy (PE) using the full time series for each channel, while dynamic BEN was derived by calculating PE within consecutive time windows. Support vector regression (SVR) was applied to predict brain age in the HC group using static and dynamic BEN features. The optimal model was then used to predict brain age in the MDD group. Finally, receiver operating characteristic (ROC) curve analysis was conducted to evaluate the discriminative capacity of BEN and brain age metrics for distinguishing MDD from HC.

**Results:**

In HC, the strongest brain age prediction was achieved by dynamic BEN derived from HbR signals (*r* = −0.62, *p* < 0.001), while in MDD, the best performance was obtained using dynamic BEN from HbO signals (*r* = −0.78, *p* < 0.001). MDD patients exhibited a significantly elevated brain age gap (BAG) compared with HC across all entropy‐based features (all *p* < 0.001). Moreover, both group‐discriminative BEN and brain age metrics derived from static and dynamic BEN achieved outstanding diagnostic performance, with several combinations reaching an area under the ROC curve of 1.00 in distinguishing MDD from HC.

**Conclusions:**

Frontal BEN derived from rs‐fNIRS represents a potential neurophysiological biomarker of accelerated brain aging in patients with MDD. Furthermore, brain age estimated from cerebrovascular hemodynamic complexity demonstrates high discriminative ability for identifying MDD. Collectively, these findings suggest that frontal neurovascular complexity metrics may serve as both diagnostic markers for MDD and quantitative indicators of pathological aging progression.

## 1. Background

Major depressive disorder (MDD) is a highly prevalent yet poorly understood mental disorder that lacks reliable objective biomarkers for clinical diagnosis [1]. Critically, accumulating evidence suggests the association of MDD with accelerated brain aging patterns that prominently affect key regions, including the frontal cortex, temporal lobe structures, limbic circuitry, and neural substrates fundamentally involved in affective regulation and higher‐order cognition [2, 3]. The neurobiological underpinnings of this association may be derived from neuroanatomical alterations observed in MDD patients, such as progressive cortical thinning and gray matter volume reduction [4], compounded by functional disruption, such as aberrant functional connectivity within brain regions related to impaired working memory, attentional dysfunction, and executive control deficits [5, 6]. Furthermore, this neuropathological cascade appears modulated by frequent psychiatric comorbidities (notably anxiety spectrum disorders) that may synergistically exacerbate brain aging processes [7]. These findings collectively highlight the urgent need to clarify the neurobiological mechanisms underlying accelerated brain aging in MDD and identify objective biomarkers for its assessment.

Brain entropy (BEN), as a measure quantifying the complexity of brain activity, has emerged as a promising candidate biomarker in neuropsychiatric disorders [8–10], including MDD [11–15]. Specifically, studies investigating BEN in MDD have revealed distinct patterns: post‐treatment decreases in BEN were observed in the posterior cerebellar lobe (PCL) of MDD patients receiving electroconvulsive therapy, alongside increased PCL‐temporal pole connectivity [11]. MDD patients with comorbid PTSD exhibited reduced BEN in specific regions, while higher BEN in the right middle frontal orbital gyrus correlated with symptom severity [16]. Smoking MDD patients demonstrated higher global BEN than non‐smokers, with network‐specific variations [12]. Decreased BEN in the bilateral thalami suggests thalamic dysfunction as a potential MDD marker [13]. Late‐life depression was associated with reduced BEN in the right posterior cingulate gyrus but increased BEN in affective processing areas [14]. Critically, BEN has been proposed as a significant diagnostic biomarker for MDD using recognition models [15]. Collectively, these findings underscore the role of altered BEN in MDD pathophysiology, offering a novel lens through which to analyze the spatiotemporal complexity of resting‐state brain activity in this disorder.

To effectively measure the hemodynamic correlates of brain activity complexity, like BEN in clinical populations, functional near‐infrared spectroscopy (fNIRS) presents distinct advantages. The fNIRS is a noninvasive neuroimaging technique that measures hemodynamic changes via near‐infrared light absorption, quantifying key biomarkers: oxyhemoglobin (HbO), indicating neural activation; deoxyhemoglobin (HbR), reflecting oxygen metabolism; and total hemoglobin (HbT), representing blood volume changes [17]. Compared to functional magnetic resonance imaging, fNIRS offers superior tolerance to motion artifacts and higher temporal resolution, while providing better spatial resolution than electroencephalography. These practical advantages position fNIRS as a highly suitable tool for clinical neuroimaging applications, particularly for investigating complex disorders like MDD. Indeed, fNIRS studies have already revealed significant alterations in MDD, such as reduced prefrontal cortex activation [18] and altered functional networks involving the middle occipital gyrus, which correlate with depressive symptoms [19]. These findings strongly indicate that resting‐state fNIRS (rs‐fNIRS) is a potentially powerful tool for investigating the pathological mechanisms underlying MDD, including those related to altered brain complexity and accelerated aging.

The present study investigates the role of BEN derived from frontal rs‐fNIRS in predicting brain age across 49 healthy controls (HCs) and 35 patients with MDD. To comprehensively characterize frontal neurovascular complexity in both temporal stability and dynamic fluctuations, we computed both static and dynamic BE measures using time‐series signals of HbO, HbR, and HbT. Spatial distributions of these BEN measures across the frontal cortex were analyzed, and their discriminative capacity for distinguishing MDD was evaluated. Finally, brain age prediction was performed using linear support vector regression (SVR). We hypothesize that (1) frontal dynamic BEN serves as an effective predictor of brain age and (2) MDD patients exhibit accelerated brain aging compared to HC.

## 2. Materials and Methods

### 2.1. Participants

This study enrolled 35 hospitalized MDD patients MDD from Shandong Dai Zhuang Hospital between October 2023 and October 2024. Diagnoses were confirmed by at least two attending psychiatrists using the International Classification of Diseases, 10^th^ Revision (ICD‐10) criteria, with all MDD patients being either medication‐naïve or maintaining stable pharmacotherapy regimens for ≥4 weeks prior to participation. Forty‐nine healthy participants in the HC group were recruited through community advertisements. The sample size was determined by power analysis (*α* = 0.05, power = 0.8) based on anticipated effect sizes in BEN metrics. Groups were matched for age, sex, and education.

To further evaluate the generalizability of the brain age prediction model, an independent validation cohort consisting of 15 healthy participants was additionally recruited using the same inclusion and exclusion criteria described later in this study.

Inclusion criteria for MDD patients comprised: (1) ICD‐10 diagnosis of MDD, (2) age 18–65 years, (3) complete at least primary school education, and (4) right‐handedness; exclusion criteria included: (1) comorbid psychiatric disorders or substance abuse/dependence history and (2) severe organic brain disorders. For HC, inclusion criteria required: (1) age 18–65 years, (2) minimum elementary education, (3) normal/corrected‐to‐normal vision, and (4) willingness to complete study procedures; exclusion criteria encompassed: (1) history of psychiatric disorders, (2) neurological or major systemic illnesses, and (3) alcohol/drug abuse or dependence history. The study protocol was approved by the Institutional Review Board of Shandong Dai Zhuang Hospital (No. 33‐2023‐202311HY‐1). Written informed consent was obtained from all participants prior to their involvement in the research.

### 2.2. Measurements

#### 2.2.1. Hamilton Depression Rating Scale‐17 Item (HAMD‐17)

The HAMD‐17 is a clinician‐administered instrument extensively utilized for assessing the severity of depressive symptoms. The scale consists of items scored on either a 0–4 or 0–2 basis, resulting in total scores ranging from 0 to 52, with higher scores indicative of more severe depression [20]. The HAMD‐17 was employed in this study to objectively measure depressive symptoms in participants diagnosed with MDD. To ensure the reliability of the assessments, evaluations were independently conducted by two experienced psychiatrists. Inter‐rater reliability was assessed using Cohen’s kappa, which produced a value of 0.58, signifying moderate agreement between the raters.

#### 2.2.2. Patient Health Questionnaire‐9 (PHQ‐9)

The PHQ‐9 is a rigorously validated self‐report instrument specifically designed to assess the severity of depressive symptoms experienced by individuals over the preceding 2‐week period. Each of the nine items on the questionnaire is rated using a 4‐point scale, ranging from 0 (“not at all”) to 3 (“nearly every day”), resulting in total scores that can range from 0 to 27 points [21]. Higher total scores are indicative of more severe depressive symptoms. The PHQ‐9 was utilized in this study as a standardized measure to evaluate depressive symptomatology in patients diagnosed with MDD.

### 2.3. The fNIRS Data Acquisition and Preprocessing

#### 2.3.1. Acquisition

**Figure 1:**
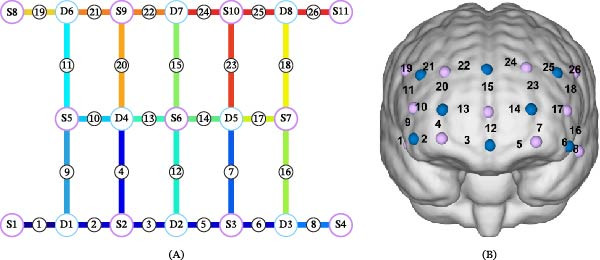
Optode arrangement of the fNIRS imaging system. (A) The system utilized an 11‐source × 8‐detector configuration generating 26 measurement channels. S indicates source optodes; D denotes detector optodes. (B) Spatial distribution of the 26 frontal cortex channels. Purple color indicates source optodes; blue color denotes detector optodes.

The rs‐fNIRS data were collected using the NirSmartII‐3000 portable system (HuiChuang Medical Technology, Danyang, China). The optical imaging system employed a configuration of 11 light‐emitting sources and 8 photodetectors arranged to form 26 measurement channels with a fixed 3 cm source‐detector separation distance (Figure 1A). This optode arrangement provided comprehensive coverage of a 30 cm × 6 cm region encompassing the frontal cortex (Figure 1B), mainly including frontopolar cortex (FPC), inferior prefrontal cortex (IPFC), temporopolar cortex (TPC), and dorsolateral prefrontal cortex (DLPFC). The system utilized dual‐wavelength near‐infrared light (730 and 850 nm) to enable simultaneous monitoring of hemodynamic changes. The brain regions corresponding to each channel were anatomically localized using Brodmann Area mapping via the Talairach Daemon system, with full details provided in the Supporting Information: Table S1.

Following a standardized experimental protocol, each participant underwent a 5‐min eye‐closed rs‐fNIRS recording session. Throughout the recording, participants were instructed to maintain a relaxed yet alert mental state while minimizing head movements to ensure optimal signal quality. The experimental environment was carefully controlled to minimize external disturbances that could affect the fNIRS measurements.

#### 2.3.2. Preprocessing

Raw fNIRS signals were processed through a standardized pipeline using NirSpark software (http://www.hcmedx.cn/). First, motion artifacts were corrected by removing signal fluctuations and spikes. Next, a 0.2 Hz low‐pass filter was applied to attenuate physiological noise (e.g., cardiac and respiratory interference) while preserving hemodynamic responses. Optical density data were then converted to oxygenated (HbO), deoxygenated (HbR), and HbT concentration changes using the modified Beer–Lambert law [22], with differential pathlength factors accounting for light scattering in brain tissue. This preprocessing approach adheres to established fNIRS guidelines, balancing noise reduction with signal fidelity. From the cleaned data, we extracted 26‐channel time‐series signals (2000 time points per channel) representing HbO, HbR, and HbT dynamics for subsequent entropy analysis.

#### 2.3.3. Signal Quality Assessment

To quantitatively evaluate fNIRS signal quality, we calculated the coefficient of variation (CV) for the raw optical intensity signals at each wavelength for every channel:

CV=σIμI,

where *μ*_*I*_ and *σ*_*I*_ represent the mean and standard deviation of the intensity time series, respectively. Channels with CV > 7.5%  were excluded from subsequent analyses to ensure an adequate signal‐to‐noise ratio [23–25].

### 2.4. BEN

We employed permutation entropy (PE) to calculate BEN. PE was chosen for its robustness to noise and amplitude variations, as it quantifies complexity based on the ordinal patterns of the time series rather than the specific amplitude values [26–29]. This property is particularly advantageous for fNIRS data, which can be affected by physiological and motion artifacts [29]. We employed PE to calculate BEN, consistent with our prior research [10, 30]. In brief, for a given time series of length *N*, the PE is calculated by embedding the series into a *d*‐dimensional space, identifying the ordinal patterns of the embedded vectors, and computing the Shannon entropy of the probability distribution of these patterns. The algorithm uses an embedding dimension *d* = 3 and a time lag *τ* = 1, consistent with established parameters for fNIRS data. For detailed mathematical formulas, please refer to Supporting Information.

#### 2.4.1. Static BEN Assessment

The static BEN was derived from the complete time series signals to assess signal complexity. For each participant, we calculated 78 static BEN measures by examining three hemodynamic time series (HbO, HbR, and HbT) across 26 predefined regions of interest (ROIs).

#### 2.4.2. Dynamic BEN Assessment

The evaluation of dynamic BEN was conducted utilizing a sliding window methodology, designed to capture fluctuations in temporal complexity, consistent with the approach employed in our prior research [10].

### 2.5. Brain Age Prediction

We conducted brain age prediction utilizing linear SVR, employing features derived from both static and dynamic BEN metrics extracted from 26‐channel fNIRS data across three hemodynamic signals: HbO, HbR, and HbT. The static BEN metrics included HbO_static (static BEN of HbO), HbR_static (static BEN of HbR), HbT_static (static BEN of HbT), and Static_all (composite static BEN). In contrast, the dynamic BEN metrics comprised HbO_dynamic (dynamic BEN of HbO), HbR_dynamic (dynamic BEN of HbR), HbT_dynamic (dynamic BEN of HbT), and Dynamic_all (composite dynamic BEN). Furthermore, we developed a comprehensive feature termed ALL, which integrated all static and dynamic BEN metrics for a holistic analysis. This multifeature approach facilitated a thorough examination of both temporal and spatial entropy characteristics in relation to brain aging.

Feature selection was performed utilizing partial Pearson correlation analysis to examine the relationship between BEN features and biological age, while adjusting for demographic covariates such as gender, age, and educational years (|*r*| > 0.1) [31].

To ensure rigorous model validation and mitigate overfitting, we employed a leave‐one‐out cross‐validation (LOOCV) framework within the HC group for each feature set (as listed in Table 1). In each iteration, one subject served as the validation set, while the remaining subjects constituted the training set. Hyperparameter tuning was performed to obtain the optimal model, with the regularization parameter C searched over [1, 10, 100, 1000]. The optimal hyperparameter was selected based on the lowest root mean square error (RMSE) and the highest correlation between biological age and predicted age in the training folds. The resulting model was then applied to the MDD test set to estimate predicted brain age. The brain age gap (BAG) was calculated as predicted brain age minus biological age. Additionally, the best‐trained model from each iteration was also applied to an independent validation cohort consisting of 15 healthy participants recruited using the same inclusion and exclusion criteria as the main HC group.

**Table 1:** The differences in the predicted brain age between HC and MDD.

Features	HC		MDD		*t*	*p*
	Mean	SD	Mean	SD		
HbO_static	27.31	0.80	33.05	1.21	−26.18	1.42E − 41
HbR_static	27.27	0.79	32.54	1.67	−19.31	2.98E − 32
HbT_static	27.87	0.89	32.80	1.45	−19.26	3.57E − 32
HbO_dynamic	27.03	0.55	32.95	1.19	−30.69	1.02E − 46
HbR_dynamic	27.41	0.74	32.96	1.78	−19.56	1.28E − 32
HbT_dynamic	27.09	0.64	32.93	1.64	−22.67	4.56E − 37
Static_all	27.54	0.73	32.77	1.34	−22.97	1.80E − 37
Dynamic_all	27.15	0.58	32.68	1.62	−22.08	2.91E − 36
ALL	27.12	0.55	32.56	1.55	−22.72	3.92E − 37

*Note: t*, two‐sample *t*‐test.

### 2.6. Statistical Analysis

Statistical analyses were performed using SPSS (version 20; IBM Corp.). Normality was assessed using Shapiro–Wilk tests. Demographic variables were compared using independent‐sample *t*‐tests or Mann–Whitney *U*‐tests for continuous data, and chi‐square tests for categorical data. Between‐group differences in static and dynamic BEN (26 channels, HbO/HbR/HbT) were analyzed using two‐sample *t*‐tests with false discovery rate (FDR) correction (*q* < 0.05). Pearson correlations were used to examine relationships between BEN and age, with FDR correction. BAG differences between HC and MDD were compared using two‐sample *t*‐tests. Diagnostic performance was evaluated using receiver operating characteristic (ROC) curve analysis, assessing individual BEN metrics (static/dynamic; HbO/HbR/HbT) and composite brain age indices.

## 3. Results

### 3.1. Characteristics of Participants

There were no statistically significant differences between the two groups concerning age, gender, years of education, or handedness (all *p*  > 0.05). MDD showed a significantly higher PHQ‐9 score than HC (Table 2). The information of independent validation cohort of 15 HC is shown in the Supporting Information: Table S3. No significant different characteristics were observed between two HC groups.

**Table 2:** Characteristics of participants.

Measurements	MDD (*n* = 35)	HC (*n* = 49)	*t/χ* ^2^	*p*
Age	33.14 ± 13.39	31.33 ± 8.73	−0.02^a^	0.98
Sex (male/female)	14/23	21/28	0.22^b^	0.64
Education	10.30 ± 3.79	11.12 ± 3.83	−1.08^a^	0.28
Handness (left/right)	37/0	49/0	0^b^	1
PHQ‐9	17.73 ± 2.9	1.14 ± 1.24	−7.98^a^	<0.001
HAMD‐17	20.34 ± 0.22	—	—	—

^a^two‐sample *t*‐test.

^b^ *χ* ^2^ test.

### 3.2. Channel Distribution of Static and Dynamic BEN

For the static BEN across 26 channels, 35 MDD showed higher values than 49 HC (Figure 2A–C,G–I). The averaged static BEN in two groups is shown in Figure 2D–F, K–M. For the dynamic BEN across 26 channels, 35 MDD showed similar values as 49 HC (Figure 2N–P,T–V). The averaged dynamic BEN in the two groups is shown in Figure 2Q–S,W–Y.

Figure 2Channel distribution values of static and dynamic BEN. (A–C) Static entropy of HbO, HbR, and HbT across 26 channels in 49 healthy controls (HC). (D–F) Distribution of mean static entropy values in HC group. (G–I) Static entropy of HbO, HbR, and HbT across 26 channels in 35 MDD patients. (K–M) Distribution of mean static entropy values in MDD group. (N–P) Dynamic entropy of HbO, HbR, and HbT across 26 channels in 49 HC. (Q–S) Distribution of mean dynamic entropy values in HC group. (T–V) Dynamic entropy of HbO, HbR, and HbT across 26 channels in 35 MDD patients. (W–Y) Distribution of mean dynamic entropy values in MDD group.

**Figure:**
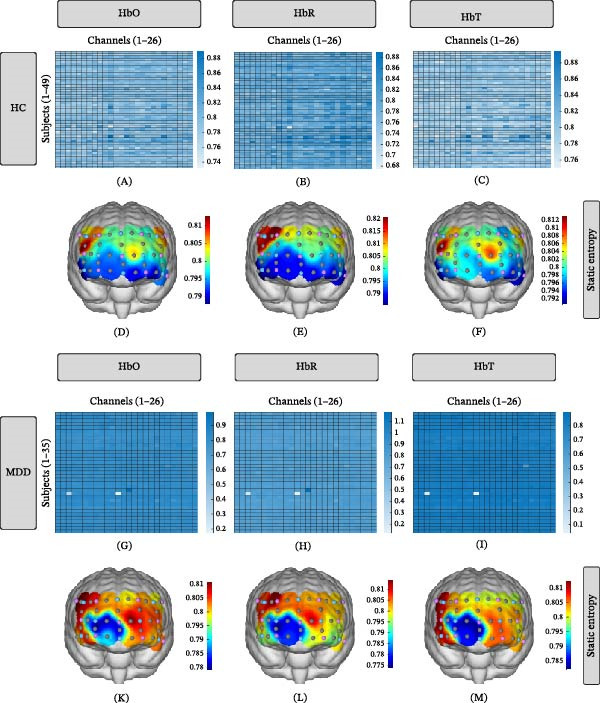

**Figure:**
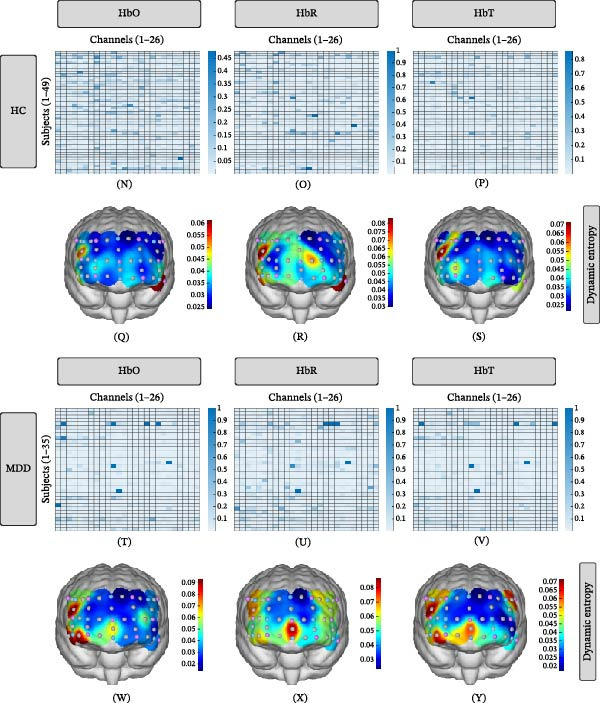

**Figure 3:**
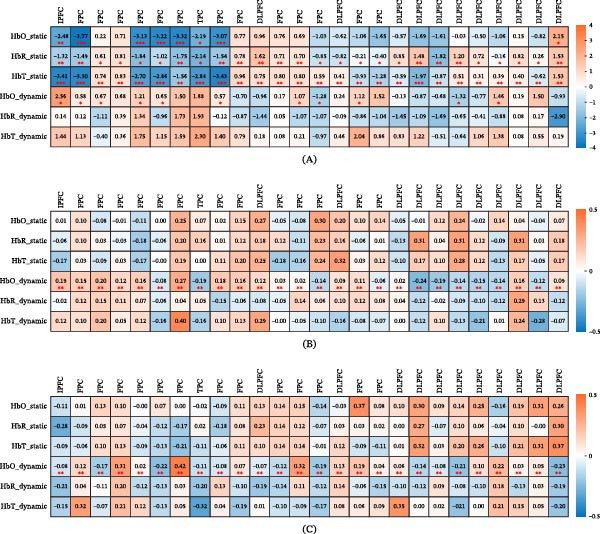
Between‐group differences and age‐related correlations of single‐channel BEN in HC and MDD patients. (A) Between‐group comparisons of BEN (two‐sample *t*‐tests). Blue: significantly lower entropy in MDD (*p* < 0.05); red: higher entropy. (B) Correlation between BEN and chronological age in HC (Pearson’s *r*). (C) Correlation between BE and age in MDD (Pearson’s *r*). Blue gradients: negative correlations (entropy decreases with age); red gradients: positive correlations (entropy increases with age). DLPFC, dorsolateral prefrontal cortex; FPC, frontopolar cortex; IPFC, inferior prefrontal cortex; TPC, temporopolar cortex. Significance levels after FDR‐corrected:  ^∗^ *p*  < 0.05;  ^∗∗^ *p* < 0.01;  ^∗∗∗^ *p*  < 0.001.

Significant differences were observed in static BEN derived from HbO, HbR, and HbT between MDD and HC (Figure 3A). Meanwhile, significant correlations between HbO‐derived dynamic BEN and chronological age in both HC and MDD groups (Figure 3B–C).

### 3.3. Brain Age Prediction Using Static and Dynamic BEN

**Figure 4:**
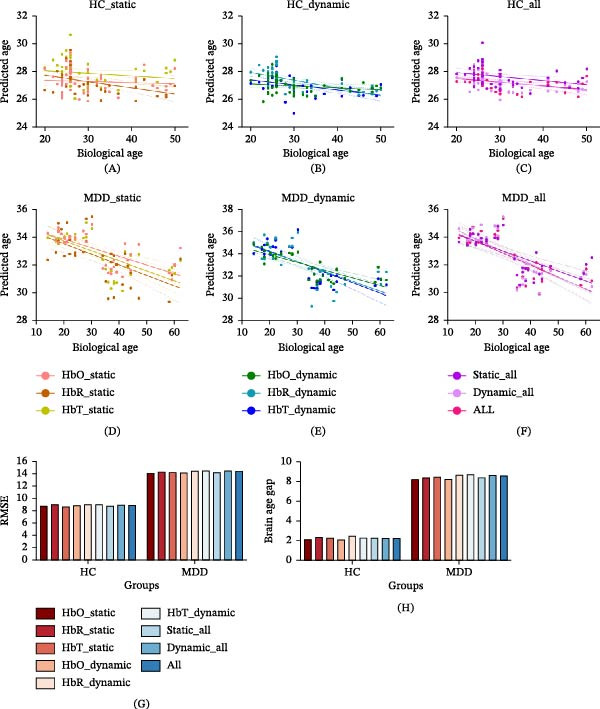
The performance of static and dynamic BEN in predicting brain age. (A) It illustrates the correlation between biological age and predicted age utilizing static BEN derived from HbO, HbR, and HbT time signals within the HC group. (B) It depicts the correlation between biological age and predicted age using dynamic BEN derived from HbO, HbR, and HbT time signals in the HC group. (C) It demonstrates the correlation between biological age and predicted age employing combinations of static, dynamic, and all BEN in the HC group. (D) It shows the correlation between biological age and predicted age using static BEN derived from HbO, HbR, and HbT time signals in the MDD group. (E) It illustrates the correlation between biological age and predicted age using dynamic BEN derived from HbO, HbR, and HbT time signals in the MDD group. (F) It represents the correlation between biological age and predicted age using combinations of static, dynamic, and all BEN in the MDD group. (G) It displays the root mean square error (RMSE) associated with brain age prediction using each type of BEN in each group. (H) It examine the brain age gap using each type of BEN in each group.

Within the HC group, the brain age predicted using dynamic BEN derived from HbR‐time signals showed the strongest correlation with biological age (Table 3 and Figure 4A–C). When this model applied to the independent cohort of 15 healthy participants, the model showed no significant difference (Supporting Information: Table S4), confirming its generalizability and the absence of systematic bias. In the MDD group, dynamic BEN based on HbO‐time signals achieved the highest correlation with biological age (Table 3 and Figure 4D–F). Group comparisons revealed that MDD patients exhibited significantly elevated BAGs (all *p*  < 0.001; Table 1 and Figure 4H).

**Table 3:** The performances of different BEN in predicting brain age.

Groups	Features	*r*	*p*	RMSE	BAG
HC	HbO_static	−0.09	0.5450	8.77	2.11
	HbR_static	−0.46	0.0009	9.04	2.36
	HbT_static	−0.17	0.2360	8.65	2.29
	HbO_dynamic	−0.21	0.1390	8.90	2.11
	**HbR_dynamic**	**−0.62**	**<0.0001**	**9.06**	2.50
	HbT_dynamic	−0.46	0.0008	9.04	2.29
	Static_all	−0.33	0.0223	8.83	2.29
	Dynamic_all	−0.43	0.0018	8.97	2.26
	ALL	−0.38	0.0071	8.94	2.26

MDD	HbO_static	−0.69	<0.0001	14.11	8.20
	HbR_static	−0.62	0.0001	14.35	8.39
	HbT_static	−0.67	<0.0001	14.27	8.45
	**HbO_dynamic**	**−0.78**	**<0.0001**	**14.20**	8.23
	HbR_dynamic	−0.67	<0.0001	14.50	8.67
	HbT_dynamic	−0.77	<0.0001	14.55	8.71
	Static_all	−0.72	<0.0001	14.25	8.39
	Dynamic_all	−0.76	<0.0001	14.52	8.65
	ALL	−0.75	<0.0001	14.46	8.58

*Note: r*, the coefficient of correlation between predicted age and biological age. Bold and underline indicate the best model with the highest r.

Abbreviations: BAG, brain age gap (predicted age − biological age); RMSE, root mean square error.

### 3.4. Discrimination of MDD Patients

**Figure 5:**
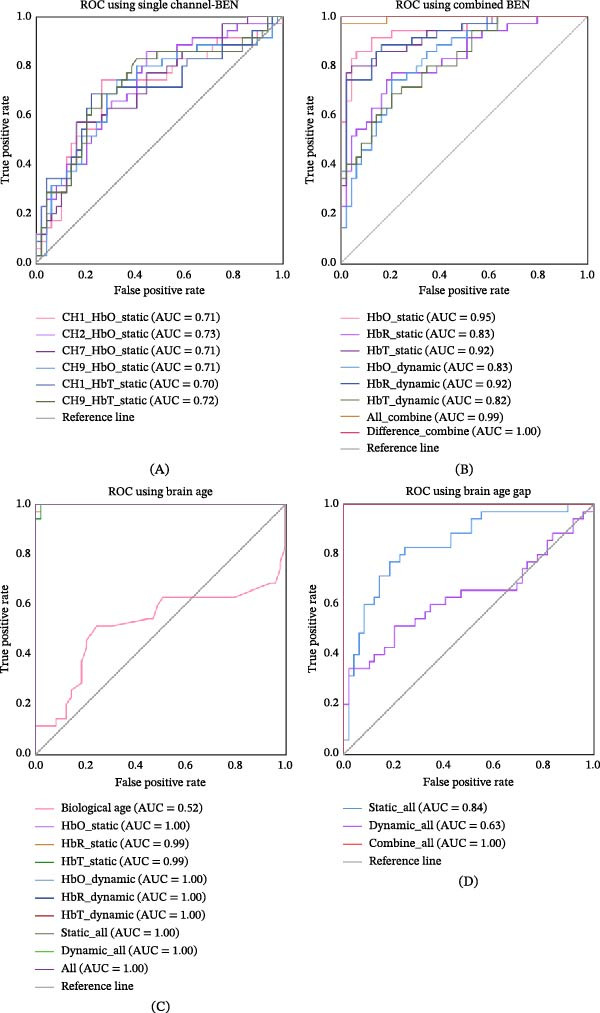
Discriminative performance of BEN and brain age metrics in MDD identification. (A) Single‐channel analysis reveals the top six most discriminative entropy features (AUC range: 0.70–0.73), with CH2_HbO_static showing the highest classification accuracy. (B) Enhanced diagnostic performance of combined hemodynamic measures, demonstrating progressive improvement from individual features (AUC = 0.82–0.95) to comprehensive combinations (AUC = 0.99–1.00). (C) Classification accuracy of brain age prediction models, with multiple feature sets achieving discrimination (AUC = 1.00). (D) Diagnostic utility of brain age gap (BAG) analysis, showing complete separation between groups (AUC = 1.00) when integrating all entropy features. AUC, area under the curve; BAG, brain age gap (predicted brain age − biological age); HbO, oxygenated hemoglobin; HbR, deoxygenated hemoglobin; HbT, total hemoglobin; MDD, major depressive disorder.

Single‐channel BEN analysis identified six features with AUC > 70% for MDD discrimination (Figure 5A). The top performers were CH2_HbO_static (AUC = 0.73), CH9_HbT_static (0.72), and four additional measures (CH1_HbO_static, CH7_HbO_static, CH9_HbO_static, CH1_HbT_static) ranging from 0.70 to 0.71. The comprehensive single‐channel BEN data for MDD discrimination are presented in the Supporting Information: Table S2. Notably, the combination of significant group‐different single‐channel BEN, as illustrated in Figure 3A, demonstrated superior performance for MDD discrimination (AUC = 1.00; Figure 5B). Other high‐performing combinations included: all‐channel entropy (0.99), HbO_static (0.95), HbT_static (0.92), and HbR_dynamic (0.92).

Brain age predicted by BEN revealed higher diagnostic accuracy than biological age (Figure 5C). Brain age predicted by HbO_static, HbO_dynamic, HbR_dynamic, and HbT_dynamic all achieved perfect classification (AUC = 1.00), while the brain age predicted by HbR_static and HbT_static showed near‐perfect performance (AUC = 0.99). Comprehensive evaluations of BAG derived from static_all (0.84), dynamic_all (0.63), and their combination (1.00) confirmed the robustness of brain age metrics in MDD identification (Figure 5D).

## 4. Discussion

The current study investigated both static and dynamic BEN derived from rs‐fNIRS signals (HbO, HbR, and HbT) for brain age prediction in HC and MDD patients. Our findings demonstrated that MDD showed accelerated brain aging. Moreover, rs‐fNIRS‐based BEN features and brain age exhibit excellent discriminative capacity for MDD identification, underscoring the potential application value of cerebrovascular hemodynamic complexity in brain aging processes and identification for MDD.

### 4.1. Distinct Predictive Power of BEN in Brain Age Prediction

Compared with static BEN in HC, the superior predictive power of dynamic BEN in health brain age prediction might originate from their unique capacity to capture the temporal complexity of neural dynamics [10, 32]. Providing a more nuanced characterization of brain state fluctuations. This temporal sensitivity parallels observations from dynamic functional connectivity research [33–37], reinforcing the importance of time‐varying neural signatures in understanding neuropsychiatric conditions. Our results align with and extend current literature documenting the clinical relevance of BEN patterns, particularly their established associations with cognitive impairment [38] and affective dysregulation [14] in psychiatric populations.

These differential prediction patterns may reflect distinct neurobiological mechanisms underlying brain aging in healthy versus depressed individuals, offering potential biomarkers for early detection and targeted intervention in mood disorders. The findings particularly highlight the value of dynamic BEN measures in capturing depression‐related alterations in neural temporal organization and hemodynamic coupling.

Furthermore, the predictive power of hemodynamic signals differed between groups. In HC, the strongest predictions were achieved with dynamic BEN derived from HbR‐time signals, whereas in MDD, HbO‐time signals were most predictive. This differential pattern may be attributed to the distinct physiological underpinnings of these signals. HbO is primarily driven by cerebral blood flow and is a proxy for neurovascular coupling and neural activation, and HbR is more directly related to oxygen extraction and metabolic demand [17]. In healthy individuals, where neurovascular coupling is intact, HbR dynamics may be a more sensitive index of metabolic efficiency, which is a key feature of normative aging. Conversely, MDD is associated with disrupted neurovascular coupling, which could alter the relationship between flow and metabolism [18, 19]. In this pathological state, HbO‐based measures of hemodynamic complexity may become more sensitive to the age‐accelerating effects of the disorder.

### 4.2. Accelerated Brain Aging in MDD

Our analyses revealed significantly elevated predicted brain age in MDD patients relative to HC, providing direct support for the accelerated brain aging hypothesis in depression [3, 39]. This finding corroborates previous reports of MDD‐related structural and functional brain changes resembling premature aging patterns [3, 39], including characteristic reductions in neuroplasticity and progressive neurodegeneration. The observed group differences in biomarker reliance may reflect underlying disturbances in oxygen metabolism efficiency or neurovascular coupling mechanisms in depression, representing promising targets for future mechanistic investigations.

### 4.3. Brain Age Demonstrates Superior Discriminative Power for MDD

Our study reveals that group‐different BEN and brain age based on fNIRS‐derived BEN metrics exhibit exceptional capability in distinguishing MDD patients from HC. This finding indicates that BEN patterns as well as brain age may serve as more sensitive and specific biomarkers of MDD than conventional hemodynamic measures alone. The superior performance of brain age prediction suggests that entropy‐based metrics capture fundamental, system‐level neural dysregulation associated with MDD, potentially reflecting the disorder’s characteristic accelerated brain aging processes. These results position BEN‐derived age estimation as a powerful neurocomputational approach for MDD identification and characterization. These results highlight the potential of fNIRS‐derived BEN as a sensitive marker for brain aging, with clinical implications for early detection and monitoring of neuropsychiatric disorders. Future studies should explore whether interventions (e.g., antidepressants or neuromodulation) can normalize these entropy patterns and mitigate accelerated brain aging in MDD.

### 4.4. Limitations

Several methodological limitations should be considered when interpreting our findings. First, while our sample size was determined by a power analysis based on effect sizes, it remains relatively modest. This limitation precludes the use of a fully independent external validation cohort for our prediction model and may affect the generalizability of our findings. Consequently, the current results should be considered preliminary, and future research with larger, multisite cohorts is necessary to validate the robustness of the brain age prediction model and its diagnostic utility. Second, our study did not account for potential confounding factors such as medication effects, comorbidities, or variations in depressive symptom severity, which could influence BEN and age prediction accuracy. Addressing these limitations in future research will strengthen the validity and generalizability of our findings. Third, given that BEN demonstrates higher sensitivity to cognitive changes and brain aging compared to other metrics, we intentionally focused on this measure without constructing alternative functional indicators for comparative brain age prediction. To address the current limitations, subsequent research ought to integrate multimodal neuroimaging markers alongside BEN to establish more robust brain age prediction models. Fourth, while our results highlight the utility of BEN, future work should optimize algorithms and recruit larger, more heterogeneous cohorts. Such efforts would clarify whether observed effects are entropy‐specific or mediated by broader functional network changes.

## 5. Conclusions

This study investigates the application of rs‐fNIRS‐derived BEN metrics to predict brain age in individuals diagnosed with MDD. The findings indicate that static BEN, as assessed through rs‐fNIRS, possesses substantial discriminative capability for identifying MDD. In contrast, dynamic BEN emerges as a critical biomarker for predicting brain age. This research enhances the comprehension of BEN dynamics in the contexts of aging and depression, underscoring the potential of rs‐fNIRS‐based dynamic measures in clinical and translational neuroscience.

## Author Contributions

Conceptualization: Hao Yu and Shanling Ji. Data collection: Xinyu Lin and Xia Liu. Methodology: Shanling Ji. Formal analysis: Shanling Ji and Yang Tian. Writing – original draft: Shanling Ji and Yang Tian. Writing – review and editing: Hao Yu and Xia Liu. Supervision: Hao Yu.

## Funding

This work was supported in part by the National Natural Science Foundation of China (Grants 82471526 and 81901358), the National Key Technology R&D Program of China (Grant 2023YFC2506204), the Natural Science Foundation of Shandong Province (Grants ZR2019BH001, ZR2021YQ55, and ZR2024QH652), and the Young Taishan Scholars of Shandong Province (Grant tsqn201909146).

## Disclosure

All content was solely created and reviewed by the authors.

## Conflicts of Interest

The authors declare no conflicts of interest.

## Supporting Information

Additional supporting information can be found online in the Supporting Information section.

## Supporting information


**Supporting Information** Supporting information includes additional method and analysis results, which include (a) the method of permutation entropy. (b) Table S1. The brain regions corresponding to each channel. (c) Table S2. The AUC of each channel‐based entropy for the discrimination of MDD. (d) Table S3. Comparison between the two HC groups. (e) Table S4. The differences in the predicted brain age between the two HC groups.

## Data Availability

The data are available upon request through the corresponding author.
