# Supplementary material for: Frontal Cortex Entropy Derived From Resting‐State fNIRS for Brain Age Prediction in Major Depressive Disorder
Source: Depress Anxiety. 2026 Apr 30;2026:4616978. doi: 10.1155/da/4616978 (PMC13129505; doi:10.1155/da/4616978)
Supplement: Supplementary file 1 — Supporting Information Supporting information includes additional method and analysis results, which include (a) the method of permutation entropy. (b) Table S1. The brain regions corresponding to each channel. (c) Table S2. The AUC of each channel‐based entropy for the discrimination of MDD. (d) Table S3. Comparison between the two HC groups. (e) Table S4. The differences in the predicted brain age between the two HC groups. [file DA-2026-4616978-s001.docx]

**Supplementary Materials**

**Supplement Method:**

### **1. Permutation entropy**

In the current study, we used the permutation entropy formula to calculate brain entropy. Permutation entropy is a modified form of Shannon entropy that assesses the complexity of ordinal patterns within a time series ^[1]^. It is supposed that the time signal T is given by formula (1):

$T=\left\{ t\left( 1 \right),t\left( 2 \right),t\left( 3 \right),\ldots,t\left( n \right) \right\}$ (1)

where $t\left( i \right)$ is the i-th time point, and n is the total number of time points. The original time series T was reconstructed, and subseries can be represented as :

$X_{i}=\left\{ x\left( i \right), x\left( i+1 \right), \ldots, x\left( i+\left( m-1 \right)*l \right) \right\}$ (2)

where *m* is the embedding dimension, and *l* is the delay time. For arbitrary *i*, the *m* number of real values $X_{i}$ can be arranged in descending order$X_{i}^{'}=\{x\left( i+\left( j_{1}-1 \right)*l \right)\leq x\left( i+\left( j_{2}-1 \right)*l \right)\leq\ldots\leq x\left( i+\left( j_{m}-1 \right)*l \right)\}$. Each point in the *m*-dimensional space, indexed by *i*, can be mapped onto one of the *m*! permutations. When each such permutation is considered as a symbol, then the reconstructed trajectory in the *m*-dimensional space is represented by a symbol sequence. The number of distinct symbols can be at most *m*!. The probability distribution for the distinct symbols is $P_{1,}P_{2,}P_{3}\ldots\ldots P_{k}$, where *k* ≤ *m*!. Permutation entropy is defined as formula (3):

$Permutation entropy=-\sum_{j=1}^{K} P_{j}lnP_{j}$, (3)

### **1.1 Static entropy**

Static entropy was constructed by using the whole time series signals. According to previous studies ^[1-4]^, we set the embedding dimension *m* = 5 and delay time *l* = 5. Static entropy was calculated as in formula (3). For each subject, we calculated 78 static entropy values as we extracted three hemodynamic time series (HbO, HbR, and HbT) across 26 predefined regions of interest (ROIs).

### **1.2 Dynamic entropy**

In dynamic brain activity assessment, such as functional connectivity, the most commonly used techniques and approaches include sliding window, coefficient of variation (CV) ^[5, 6]^. CV is a statistical measure used to quantify the relative variability of a dataset over time.

In this study, for each ROI, the whole time-series signals were divided into segments with a window length =50TRs and an overlapping ratio of 0.9. Then, the entropy value was computed for each time segment using formula (1). The mean value ($E_{mean}$) and standard deviation ($E_{SD}$) of entropy across all time segments were computed as formula (4) and formula (5):

$E_{mean}=\sum_{t=1}^{n} E_{t}/n$ (4)

$E_{SD}=\sqrt{\frac{\sum_{t=1}^{n} \left( E_{t}-E_{mean} \right)^{2}}{n}}$ (5)

where $E_{t}$ is the entropy of the *t-*h time segment, and *n* is the total number of time segments. $E_{mean}$ represents the mean entropy across all time segments and $E_{SD}$ represents the SD. Finally, we estimated the ${CV}_{entropy}$ as the measurement of dynamic brain entropy as formula (6):

${CV}_{entropy}={E_{SD}/E}_{mean}$ (6)

**References**

[1] Bandt C, Pompe B. Permutation entropy: a natural complexity measure for time series [J]. Phys Rev Lett, 2002, 88(17): 174102.

[2] D. Jordan RI, V. Riedl, A. Schorer, S. Grimberg, S. Neufang. Electroencephalographic Order Pattern Analysis for the Separation of Consciousness and Unconsciousness [J]. Anesthesiology, 2013, 119: 1031-1042.

[3] Thul A, Lechinger J, Donis J, et al. EEG entropy measures indicate decrease of cortical information processing in Disorders of Consciousness [J]. Clin Neurophysiol, 2016, 127(2): 1419-1427.

[4] Cao Y, Tung WW, Gao JB, et al. Detecting dynamical changes in time series using the permutation entropy [J]. Phys Rev E Stat Nonlin Soft Matter Phys, 2004, 70(4 Pt 2): 046217.

[5] Engel AK, Gerloff C. Dynamic functional connectivity: causative or epiphenomenal? [J]. Trends Cogn Sci, 2022, 26(12): 1020-1022.

[6] Jalilianhasanpour R, Ryan D, Agarwal S, et al. Dynamic Brain Connectivity in Resting State Functional MR Imaging [J]. Neuroimaging Clin N Am, 2021, 31(1): 81-92.

**Supplement Tables:**

**Table S1.** The brain regions corresponding to each channel

| **Channel Number** | **Brodmann Area** | **Abbreviations** |
| --- | --- | --- |
| CH1 (S1-D1) | 47 - Inferior prefrontal gyrus | IPFC |
| CH2 (S2-D1) | 10 - Frontopolar area | FPC |
| CH3 (S2-D2) | 10 - Frontopolar area | FPC |
| CH4 (S2-D4) | 10 - Frontopolar area | FPC |
| CH5 (S3-D2) | 10 - Frontopolar area | FPC |
| CH6 (S3-D3) | 10 - Frontopolar area | FPC |
| CH7 (S3-D5) | 10 - Frontopolar area | FPC |
| CH8 (S4-D3) | 38 - Temporopolar area | TPC |
| CH9 (S5-D1) | 10 - Frontopolar area | FPC |
| CH10 (S5-D4) | 10 - Frontopolar area | FPC |
| CH11 (S5-D6) | 9 - Dorsolateral prefrontal cortex | DLPFC |
| CH12 (S6-D2) | 10 - Frontopolar area | FPC |
| CH13 (S6-D4) | 10 - Frontopolar area | FPC |
| CH14 (S6-D5) | 10 - Frontopolar area | FPC |
| CH15 (S6-D7) | 9 - Dorsolateral prefrontal cortex | DLPFC |
| CH16 (S7-D3) | 10 - Frontopolar area | FPC |
| CH17 (S7-D5) | 10 - Frontopolar area | FPC |
| CH18 (S7-D8) | 9 - Dorsolateral prefrontal cortex | DLPFC |
| CH19 (S8-D6) | 9 - Dorsolateral prefrontal cortex | DLPFC |
| CH20 (S9-D4) | 9 - Dorsolateral prefrontal cortex | DLPFC |
| CH21 (S9-D6) | 9 - Dorsolateral prefrontal cortex | DLPFC |
| CH22 (S9-D7) | 9 - Dorsolateral prefrontal cortex | DLPFC |
| CH23 (S10-D5) | 9 - Dorsolateral prefrontal cortex | DLPFC |
| CH24 (S10-D7) | 9 - Dorsolateral prefrontal cortex | DLPFC |
| CH25 (S10-D8) | 9 - Dorsolateral prefrontal cortex | DLPFC |
| CH26 (S11-D8) | 9 - Dorsolateral prefrontal cortex | DLPFC |

Note: Brodmann Area (Talairach daemon)

**Table S2.** The AUC of each channel-based entropy for the discrimination of MDD

| Channel number | Static entropy | | | Dynamic entropy | | |
| --- | --- | --- | --- | --- | --- | --- |
|  | HbO | HbR | HbT | HbO | HbR | HbT |
| CH1 | 0.706 | 0.587 | 0.704 | 0.396 | 0.520 | 0.431 |
| CH2 | 0.726 | 0.647 | 0.697 | 0.511 | 0.553 | 0.413 |
| CH3 | 0.656 | 0.606 | 0.617 | 0.371 | 0.476 | 0.482 |
| CH4 | 0.629 | 0.612 | 0.598 | 0.512 | 0.521 | 0.500 |
| CH5 | 0.664 | 0.607 | 0.668 | 0.319 | 0.464 | 0.347 |
| CH6 | 0.676 | 0.532 | 0.676 | 0.449 | 0.572 | 0.479 |
| CH7 | 0.710 | 0.630 | 0.651 | 0.447 | 0.342 | 0.437 |
| CH8 | 0.639 | 0.643 | 0.683 | 0.464 | 0.461 | 0.381 |
| CH9 | 0.707 | 0.624 | 0.722 | 0.502 | 0.494 | 0.444 |
| CH10 | 0.631 | 0.609 | 0.589 | 0.570 | 0.494 | 0.448 |
| CH11 | 0.454 | 0.401 | 0.490 | 0.559 | 0.560 | 0.441 |
| CH12 | 0.571 | 0.629 | 0.581 | 0.413 | 0.468 | 0.445 |
| CH13 | 0.642 | 0.634 | 0.615 | 0.531 | 0.576 | 0.520 |
| CH14 | 0.556 | 0.492 | 0.494 | 0.533 | 0.513 | 0.529 |
| CH15 | 0.558 | 0.564 | 0.507 | 0.527 | 0.577 | 0.441 |
| CH16 | 0.535 | 0.539 | 0.567 | 0.405 | 0.438 | 0.343 |
| CH17 | 0.585 | 0.515 | 0.625 | 0.442 | 0.589 | 0.487 |
| CH18 | 0.528 | 0.428 | 0.545 | 0.479 | 0.603 | 0.515 |
| CH19 | 0.640 | 0.453 | 0.650 | 0.377 | 0.521 | 0.460 |
| CH20 | 0.583 | 0.647 | 0.551 | 0.549 | 0.601 | 0.539 |
| CH21 | 0.528 | 0.476 | 0.572 | 0.622 | 0.534 | 0.519 |
| CH22 | 0.544 | 0.513 | 0.515 | 0.536 | 0.477 | 0.378 |
| CH23 | 0.585 | 0.518 | 0.503 | 0.438 | 0.496 | 0.453 |
| CH24 | 0.487 | 0.439 | 0.456 | 0.470 | 0.487 | 0.409 |
| CH25 | 0.543 | 0.489 | 0.499 | 0.406 | 0.405 | 0.500 |
| CH26 | 0.370 | 0.430 | 0.433 | 0.529 | 0.650 | 0.475 |
| Combine | 0.945 | 0.829 | 0.923 | 0.829 | 0.922 | 0.817 |

***Note****:* AUC, Area Under the Curve.

**Table S3.** Comparison between the two HC groups

| Measurements | HC (n＝49) | HC (*n*＝15) | *t/χ^2^* | *p* |
| --- | --- | --- | --- | --- |
| Age | 31.33±8.73 | 30.21±6.53 | -0.01 ^a^ | 0.98 |
| Sex (Male/Female) | 21/28 | 8/7 | 0.51 ^b^ | 0.48 |
| Education | 11.12±3.83 | 12.07±4.00 | -0.70 ^a^ | 0.68 |
| Handness (left/Right) | 49/0 | 15/0 | 0 ^b^ | 1 |

***Note:*** ^a^, independent samples t-test; ^b^, χ² test;

**Table S4.** The differences in the predicted brain age between the two HC groups

| Features | HC (n＝49) | |  | HC (n＝15) | | t | p |
| --- | --- | --- | --- | --- | --- | --- | --- |
|  | Mean | SD |  | Mean | SD |  |  |
| HbO_static | 27.31 | 0.80 |  | 28.80 | 6.16 | 0.82 | 0.42 |
| HbR_static | 27.27 | 0.79 |  | 27.27 | 0.77 | 0.17 | 0.86 |
| HbT_static | 27.87 | 0.89 |  | 27.39 | 0.62 | -0.52 | 0.61 |
| HbO_dynamic | 27.03 | 0.55 |  | 27.71 | 0.89 | 0.61 | 0.55 |
| HbR_dynamic | 27.41 | 0.74 |  | 27.08 | 0.49 | -0.30 | 0.77 |
| HbT_dynamic | 27.09 | 0.64 |  | 27.56 | 0.72 | -0.66 | 0.51 |
| Static_all | 27.54 | 0.73 |  | 27.29 | 0.55 | -1.14 | 0.26 |
| Dynamic_all | 27.15 | 0.58 |  | 27.55 | 0.61 | -0.07 | 0.94 |
| ALL | 27.12 | 0.55 |  | 27.33 | 0.51 | -1.13 | 0.26 |

***Note****:* t, two-sample t-test.
